# Trust in public health policy in the time of the COVID-19 epidemic in Israel

**DOI:** 10.1186/s13584-024-00607-x

**Published:** 2024-04-25

**Authors:** Jochanan Benbassat

**Affiliations:** Department of Health Policy Research, Myers-JDC-Brookdale Institute, Jerusalem, Israel

**Keywords:** COVID-19, Medical error, Public trust, Professional opinion

## Abstract

The government of Israel provides universal health care through four health care organizations (“sick funds”) that enjoy general public trust. In hindsight, the response of the government to the COVID-19 epidemic seems reasonable. In the first year of the epidemic, tests and vaccines were developed and other measures were taken, including social distancing, focusing on risk factors for infection and disease severity, and improving treatment. The COVID-19 mortality rate between January 2000 and June 2021 was around 750 per million inhabitants, well below the OECD average of 1300. Still, although the control measures were largely well received, the media and an ad hoc non-governmental Emergency Council for the coronavirus crisis in Israel criticized the government’s response to the epidemic thereby contributing to a decline in public trust in government policy. This commentary provides an overview of the importance of trust in medical institutions and the difficulties of evaluating healthcare decisions in an attempt to justify three conclusions. First, when physicians and self-appointed experts publicly disapprove of a government policy, they should consider the trade-off between improving care and undermining public trust. Second, when evaluating a medical decision, experts should not ask, “Would I have acted differently?” but rather, “Was the decision under review completely unreasonable?” Thirdly, criticism is certainly worth listening to. However, I believe that by calling for organized resistance against the government, the publicly announced establishment of the Emergency Council for the Corona crisis blatantly crossed the line between constructive criticism and destructive mistrust.

**Supplementary Information:**

The online version contains supplementary material available at 10.1186/s13584-024-00607-x.

## Background

The first COVID-19 case in Israel was diagnosed on February 21, 2020; within a month, the total number of cases reached 4,000. The Ministry of Health imposed a lockdown, which probably helped to reduce the daily infection rate to about 100 new cases on April 30, 2020, and to 20–40 cases in May [19]. However, the number of cases increased again at the end of May and reached a total of more than 2,000 in June. This “second wave” continued until October 2020, followed by a third wave in December 2020-February 2021 and a fourth wave in August-September 2021 [15]. Between March 2020 and October 2021, COVID-19 mortality in Israel totaled 8,095 [17], and mortality per million population between January 2020 and June 2021 was around 750, well below the OECD average of around 1,300 [8].

Uncertainty about the epidemic sparked controversy. Many opinions were expressed on topics such as travel restrictions, social distancing, wearing masks, and testing. Eight months after the first COVID-19 case in Israel, reports in the media were described as “chaotic”, with opinions varying widely among politicians, health administrators, doctors, physicists, and economists [10]. Some of them made witty remarks such as “Corona is a flu with public relations”; others predicted different mortality rates [5]; still others disagreed about the vaccine’s effectiveness [18]. One expert pointed out that the lockdown was more severe than in most developed countries and suggested that mitigation rather than containment of the epidemic would achieve similar results while reducing the economic impact of the containment approach [4].

Finally, a group of leading doctors, researchers, and social workers founded an ad-hoc non-governmental Public Emergency Council for the Corona Crisis. Their website (www.pecc.org.il) states: “Given the way the COVID-19 crisis is being handled [by the government],…. the members of the Council can no longer remain silent”, and that “… they bring decades of experience and knowledge — each in their field—thus creating a synergy and a balanced vision that considers the virus alongside other aspects of physical and mental health and social welfare. This vision has been sorely lacking in the current crisis management, and the Council is committed to acting as a professional body capable of formulating and advancing broader… solutions”.

The Council’s website described it as voluntary and apolitical. It solicited donations and included an appeal to an unspecified audience to join the Council. It opposed lockdowns and recognized the safety of the vaccine and its importance in combating the epidemic but rejected coercion. The Council recommended opening schools, balancing the risk of COVID-19 infection against other risks such as medical neglect, economic collapse, depression, and isolation, and helping people focus on sources of strength, hope, and success while avoiding intimidation, threats, and negative messages.

The spread of information about the COVID-19 pandemic also led to discussions about its ethical aspects. Guttman and Lev [14] focused on four issues and illustrated each of them with examples from Israeli news articles. These issues were first, the harm of using fear tactics, secondly by the promotion of stigmatization and ageism, thirdly by the disclosure of individuals’ personal information, and fourthly by the blurring of authorities’ responsibility and emphasis on values such as personal responsibility and caring for others [14]. In this paper, I propose another ethical issue, namely the impact of news media criticism on public trust.

Even if appropriate, this criticism contributed to a decline in public trust in government policy. Three months after the first wave, a survey found that health professionals had only moderate confidence in the national COVID-19 health policy [34]; surveys during the first year of the epidemic found that only 24% of Israelis agreed with the government’s crisis management [28] and fluctuations in risk perception and trust were found to affect compliance with regulations [6]. Finally, between November 1, 2020, and February 18, 2021, a poll found that the public supported the government’s policy on vaccinations, but not on lockdowns [12].

This commentary emphasizes the importance of trust in healthcare institutions. It addresses the difficulties of evaluating medical decisions and the dividing line between constructive criticism and destructive mistrust. No attempt is made to compare what happened with the various predictions or to analyze the credibility, evidence, and logic behind the criticism of individual experts or the Public Emergency Council on the Corona crisis.

## Trust: definition and importance

Trust in people or institutions is the expectation that they will protect the interests of the trustee when necessary. It allows the individual to rely on another person to make decisions that align with their interests. Because trust can influence an individual’s decision-making [2], it is the foundation of healthcare in general and healthcare crisis management in particular [20]. Trust is also the “glue” that keeps society running by preventing mistrust and excessive legislation. Distrust of doctors and medical institutions harms collaboration. It encourages litigation and defensive medicine and forces the healthcare system to divert some of its resources from treatment to self-protection.

Israel introduced a national health insurance scheme in 1996, in which all residents are insured. Services are provided by four Health Maintenance Organizations (HMOs), which enjoy the trust of the public [9]. Patients place their trust in institutions (social trust) and doctors (personal trust) [23]. Social trust is determined by prevailing opinion and media portrayal. Hence the importance of a 2019 study that found that misinformation in the media is widespread and usually characterized by negative tones that trigger distrust [32]. Timely and accurate risk communication by those responsible is crucial in emergencies, as it determines whether the public trusts the authorities more than rumors and misinformation [20]. Personal trust is determined by the patient’s experience and the physician’s perceived competence and interpersonal skills [25]. It is higher than social trust because patients ultimately find a doctor they trust [16].

These opposing objects of trust can influence each other. First, a halo effect can extend patients’ trust in their doctors to trust in the hospital or health insurance company [13]. Second, institutional trust can influence individual trust [7, 24]. This is especially true for newly formed relationships: If one knows little about a new physician, one is likely to base the relationship on general attitudes toward the employing institution [16, 24].

In Israel, public trust and response to guidelines was one of three COVID-19-related topics discussed at the 20th annual gathering of health experts in May 2021 [30]. It was acknowledged that public confidence in government guidelines declined since the start of the epidemic. This decline was attributed to the limited success in providing clear information to the public, the lack of a unified policy for dealing with the epidemic, the frequent changes in guidance, the insufficient involvement of experts in policy formulation, the multiplicity of stakeholders such as politicians, economists, local authorities as well as the Ministry of Health, hospitals and health insurers, and the increasing public perception of the risk of the disease [27].

Similarly, an international survey conducted between March 20 and April 8, 2020, concluded that public confidence in the government requires, first, health measures to control the epidemic and economic relief. Secondly, fair and effective accountability of government authorities. Third, the provision of impartial, transparent, and truthful government communication [22]. Other authors suggested establishing independent or government-managed national disease outbreak centers that provide expertise; soliciting information and advice from health experts, researchers, and international colleagues; maintaining connection with those affected by decisions, and offering a credible vision for the future; taking responsibility and doing so visibly [1].

Professional criticism is necessary, even if it can undermine public trust in institutions. It permits an evaluation of medical decisions in general and those in public health. Investigating perceived errors is critical to maintaining the quality of care, as it can lead to rethinking the process and making the healthcare system as error-resistant as possible. However, as described in the next section, it is difficult to detect errors.

## Barriers to the assessment of medical and public health decisions

Medical decisions require predictions in terms of probabilities and not in terms of right/wrong. Although reducible, uncertainty in predictions is unavoidable. I believe that some of the critics of government policy have not only ignored the trade-off between the utility of their analysis and the threat to public confidence but that they should have been more cautious in their criticism given the three difficulties in evaluating medical decisions.

The first is the difficulty of reconstructing the context of a supposedly erroneous decision. Today, it is increasingly recognized that medical decision-making is not only a cognitive endeavor but also a contextually and socially mediated activity that can be influenced by the circumstances of the decision. This influence is also changing our understanding of decision-making and the definition of medical error. As recently as 2022, it was noted that “context specificity… can free us from the search for singular definitions of expertise” [33].

A second difficulty arises from the low to medium reliability of expert judgments [21]. Reliability is the extent to which two or more experts make the same judgment about the same scenario. For example, Brennan et al. (1991) compared the judgments of two experts reviewing a sample of 318 hospital records. They agreed that there was no negligence in 293 of the records, but found negligence in the remaining 25 records. However, the two experts agreed in only four of these 25 cases. In the remaining 21 (sic! ), only one expert found medical negligence, while the other did not.

The third difficulty arises from the tendency to believe that “the writing was on the wall.” This difficulty does not apply to the criticism voiced during the first COVID-19 wave. It may, however, apply to later criticism of the government’s response to the epidemic. The presence of hindsight bias was documented by Fischhoff in 1975 [11]. He presented physicians with a clinical scenario and asked them to rate the likelihood of each possible diagnosis. Some physicians were presented only with the scenario; others were given the same scenario along with information about the final diagnosis, but were asked to ignore this information when evaluating the likelihood of the diagnosis. The comparison between the two groups showed that prior knowledge of the final diagnosis doubled the average assessment of probability. Fischhoff called the phenomenon “creeping determinism”: although participants were instructed to ignore the final diagnosis, they could not free themselves from the tendency to give more weight to the parts of the scenario that fit the final diagnosis. In other words, once we know the outcome, we can no longer objectively judge the behavior that led to that outcome.

## Conclusions

The World Health Organization defines a medical error as a caregiver’s action that deviates from the accepted standard. However, in the case of the COVID-19 epidemic, there was no evidence to justify setting a medical standard, and the appropriate response to the epidemic was wrought with uncertainties. Nevertheless, I believe that the data presented justify three conclusions.

First, when disapproving of a government policy through the media, experts should consider the trade-off between improving policy and undermining public trust in institutions. One of the biggest challenges in evaluating health policy is to draw the line between constructive criticism and destructive mistrust. On the one hand, criticism is necessary because health systems are not immune to mistakes, and those responsible should be prepared to answer questions and admit missteps and failures [3]. On the other hand, it has been argued that the middle of the pandemic is not the right time to recognize the weaknesses of those responsible [26]. This is because the economic upheaval caused by restrictive public health measures will inevitably provoke resistance [1], and to cooperate with these measures, the community must trust its leadership [29].

Second, given the difficulties in evaluating medical decisions that I described earlier, experts who are asked to evaluate the performance of a colleague should endeavor to reconstruct the circumstances of the event and humbly remember that their judgments can be less than reliable. Therefore, I suggest that experts apply the test of reasonableness: Instead of asking, “Would I have acted differently?” they should ask, “Was the decision under review completely unreasonable?” I suggest that critics of public health institutions should similarly apply the test of reasonableness when drawing the line between constructive criticism and destructive mistrust. When deciding how, when, where, and with whom to share their criticism, they should not ask, “Would I have made a different decision?” but rather, “Was the decision (of the government, of the institution) completely unreasonable?”

Thirdly, I believe that a distinction should be made between the criticism by individuals and ad hoc groups such as the Public Emergency Council for the Corona Crisis in Israel. Even if the criticism of this council turns out to be correct, its very creation blatantly crossed the line between constructive criticism and destructive mistrust by calling for an *organized* movement against the government and the undermining of its authority. In and of itself, this call casts doubt on the Council’s self-description as “apolitical”.

This latter conclusion should not be interpreted as opposing non-governmental organizations (NGOs) in general. NGOs aim at improving, e.g., social conditions or protecting the environment. In the U.S., there are about 1.5 million NGOs, and they are supported by the government [31]. In the UK, an example of an appropriate NGO is the Scientific Advisory Group for Emergencies (SAGE) (https://www.gov.uk/government/organisations/scientific-advisory-group-for-emergencies/about). It called for transparency in the COVID-19 policy and provided scientific and technical *support* to government decision-makers during emergencies. *I believe that the dividing line between appropriate and inappropriate NGOs is the explicit call by the latter for an organized movement against government decisions on a specific subject despite the absence of evidence that the decisions of the government were completely unreasonable. I feel that the uncertainty in the early days of the coronavirus epidemic warranted a more modest and restrained criticism than was implied on the Emergency Council’s website.*

### Electronic supplementary material

Below is the link to the electronic supplementary material.

Supplementary Material 1

Supplementary Material 2

## Data Availability

Not applicable.
