# Supplementary material for: Trust in public health policy in the time of the COVID-19 epidemic in Israel
Source: Isr J Health Policy Res. 2024 Apr 25;13:24. doi: 10.1186/s13584-024-00607-x (PMC11044392; doi:10.1186/s13584-024-00607-x)
Supplement: Supplementary file 1 — Supplementary Material 1 [file 13584_2024_607_MOESM1_ESM.docx]

March 21, 2024

Prof A. Israeli

Editor

Israel Journal of Health Policy Research

Dear Professor Israeli

Following your last request, I am resubmitting the third revision of the commentary "TRUST IN PUBLIC HEALTH POLICY IN THE TIME OF THE COVID-19 EPIDEMIC" and responding to the reviewer's comments to my former revision of this paper. The changes in the text of the first revision are in red. Please see my responses (in *ITALICS)* to the additional comments by the reviewers (in **BOLD** Letters).

Sincerely,

Jochanan Benbassat MD
