# Supplementary material for: Trust in public health policy in the time of the COVID-19 epidemic in Israel
Source: Isr J Health Policy Res. 2024 Apr 25;13:24. doi: 10.1186/s13584-024-00607-x (PMC11044392; doi:10.1186/s13584-024-00607-x)
Supplement: Supplementary file 2 — Supplementary Material 2 [file 13584_2024_607_MOESM2_ESM.docx]

Reviewer #3:

**1. The author has taken into account some of my suggestions. I appreciate that he did not agree with all my comments.  Again, I think the discussion of trust is generally well done**

*I thank the reviewer for this comment*

**2. A broader discussion of non-governmental, volunteer, and self-appointed activists or committees (such as the Independent SAGE) and their role in fostering or undermining trust is warranted to make this commentary generalizable (and not just a critique of a local group). Would the formation of any such group "cross the line" in his view?**

*I apologize for my previous inappropriate response to the same comment in an earlier version of this paper. As I wrote in my response,* *I do not oppose the establishment of non-governmental professional groups, such as the SAGE in the UK that called for enhanced transparency of the COVID policy and support the government response to the pandemic. I oppose only those who call explicitly for an organized movement to oppose the government and undermine its authority. Please see the final paragraph of the conclusions in response to this comment*

**3. He writes:" The mere establishment of the council blatantly crossed that line by calling for an organized movement to oppose the government and undermine its authority". When is opposition warranted and when does it cross the line, especially, as the author notes, when non-professionals are given a large amount of weight in government decision-making?**

*I feel that I responded adequately to this comment in paragraph 3 of the CONCLUSIONS*

Editor note:

**Please add at the end of your commentary something like: the views presented in this commentary are not necessarily those of the editors or reviewers of the IJHPR.**

*Done*

Top of Form

Bottom of Form
